# Micronutrient status of Palestinian school children following salt and flour fortification: a cross-sectional study

**DOI:** 10.1186/s40795-020-00367-2

**Published:** 2020-08-26

**Authors:** Salwa Massad, Mehari Gebre-Medhin, Omar Dary, Marwah Abdalla, Steve Holleran, Wahida Karmally, Paula Bordelois, Umaiyeh Khammash, Richard J. Deckelbaum

**Affiliations:** 1 Juzoor for Health and Social Development, Ramallah, 970 Palestine; 2 Department of Women’s and Children’s Health, Pediatrics, University Hospital, SE-751 85 Uppsala, Sweden; 3 USAID Bureau of Global Health, Washington, DC USA; 4 Division of Cardiology, Department of Medicine, Columbia University Irving Medical Center, New York, NY 10032 USA; 5 Institute of Human Nutrition, Columbia University, New York, NY 10032 USA; 6 Irving Institute for Clinical and Translational Research, Columbia University Irving Medical Center, New York, NY 10032 USA; 7 Mailman School of Public Health, Columbia University Irving Medical Center, New York, NY 10032 USA; 8 Department of Pediatrics, Vagelos College of Physicians and Surgeons, Columbia University Irving Medical Center, New York, NY 10032 USA

**Keywords:** Palestine, Children, Micronutrient, Anemia, Fortification

## Abstract

**Background:**

In 1996 and in 2006, Palestine initiated salt iodization and multiple micronutrient fortification of wheat flour, respectively as a strategy to prevent deficiencies of these nutrients. In 2009, we assessed the impact of these interventions on the health and nutritional status of schoolchildren residing in the West Bank.

**Methods:**

We surveyed a sample of 22 schools run by the UN Relief and Works Agency for Palestine Refugees in the Near East (UNRWA) and the Palestinian Government. We randomly selected students from the first (mean age 6.7 years [SD 0.5]), sixth (11.8 years [0.6]), and ninth grades (14.8 years [0.6]). Data were obtained from 1484 (99%) of 1500 students planned for enrollment.

**Results:**

Our results suggest that iodine intake appears adequate and there was essentially no iodine deficiency. As to the status of other micronutrients, the main nutritional micronutrient risks for schoolchildren in the West Bank continue to be low serum levels of iron, zinc, and vitamin B-12; folate levels were seemingly high. The overall prevalence of anemia was 9.6%, but there were pockets of anemia in certain districts. Almost 42% of the anemia in our sample was explained by iron deficiency. There were significant differences in iron deficiency between girls and boys, 29.5% vs. 15.7%, respectively (*p* = 0.0001). There were no cases of lead toxicity in the studied sample.

**Conclusions:**

Wheat flour and salt fortification has had a major influence on improving the micronutrient status of Palestinian children, for some but not all micronutrients. The recommended key blood and biochemical parameters to be incorporated in the surveillance system are iron, zinc, and vitamin B12.

## Background

Micronutrient deficiencies are a major global health problem. Vitamin A, iron, and zinc deficiencies have the largest remaining disease burden among the micronutrients considered [1]. Most people with micronutrient deficiencies live in low income countries and are typically deficient in more than one micronutrient [2]. While adequate nutritional status is an obvious building block of human capital, it is paramount in the early stages of life. If early life nutritional demands are not met, the far-reaching and long lasting consequences on both individuals and society include: poorer adult health, lower educational attainment, a diminished work capacity, and ultimately a lower lifetime earning potential [3].

Micronutrient deficiencies are among the key nutrition challenges facing the Eastern Mediterranean Region (EMR) and the Arab world. Several micronutrient deficiencies including low levels of iron, iodine, zinc, calcium, folate, and vitamins A and D are still being reported from many countries of EMR, particularly among children and women of childbearing age [4]. When compared to other developing countries, anemia in the Arab world appears to be a moderate public health problem, with prevalence ranging from 20 to 40% [5]. The most common type of anemia in all Arab countries is iron deficiency anemia; about 50% of anemia cases are due to iron deficiency, although this proportion varies between different population groups and regions [5].

As of 2017, 4.78 million Palestinians lived in the State of Palestine. Of those, 2.99 million resided in the West Bank and 1.99 million resided in the Gaza Strip. Of the total Palestinian population, 41% are refugees. 26% of the Refugee population lives in the West Bank while the remaining 64% reside in the Gaza Strip [6]. As of 2013, 45% of the total Palestinian population was composed of children, with 43% of them living in the West Bank and 48% in the Gaza Strip [7]. Restrictions on the movement of Palestinian goods and people were imposed in September 2000 following the second intifada (uprising), affecting movement both across borders and within the Palestinian Territory. These restrictions have been accompanied by an increase in the rate of stunting in children under the age of 5, where the prevalence in the West Bank has risen from 7% in 1996 to 11% in 2010 [8]. Moreover, 33% of Palestinian households were food insecure in 2010 [9].

In Palestine, as a strategy to combat micronutrient deficiencies, salt iodization was initiated in 1996 [10], and wheat flour was fortified with eight vitamins (vitamins A, D, B-1, B-2, niacin, B-6, folic acid, and B-12), and 2 trace minerals (iron and zinc) in 2006. The flour fortification formula is shown in Table 1. Wheat flour is widely consumed in Palestine in the form of bread and is affordable to groups vulnerable to micronutrient deficiencies [10].

**Table 1:** Palestinian Flour Fortification Formula (Palestinian National Authority, Ministry of Health, Primary Health Care & Public Health Directorate, 2015)

	Average addition level	Minimum-maximum level^a^
Iron (ferrous sulfate) (mg/kg)	34.4	25.0–60.0
Zinc (zinc oxide) (mg/kg)	20.6	15.0–40.0
Folic acid (mg/kg)	1.5	1.0–2.5
Vitamin B12 (μg/kg)	4.0	min. 2.5
Thiamine (mononitrate) (mg/kg)	2.9	min. 2.0
Riboflavin (mg/kg)	3.6	min. 2.5
Vitamin B6 (pyridoxine) (mg/kg)	3.6	min. 2.5
Niacin (niacinamide) (mg/kg)	35.0	min. 25.0
Vitamin A (palmitate, CWS-250) (mg/kg)^b^	1.5	1.00–2.5
Vitamin D_3_ (100 CWS/A) (mg/kg)^b^	0.023	0.015–0.050

^a^Minimum and safe maximum levels of fortification

^b^*CWS* cold water soluble, *CWS/A* cold water soluble, coated w. gum Arabic

Despite the plethora of studies from other regions of the world, there is a paucity of data on micronutrient deficiency in Palestine overall. Previous studies on micronutrient deficiencies in Palestine were conducted mostly in small groups in the West Bank and Gaza Strip among preschool children in marginalized areas, adolescents in north Gaza and two districts in the West Bank, and pregnant women attending the United Nations Relief and Works Agency for Palestine Refugees in the Near East (UNRWA) clinics, where almost all surveyed only anemia prevalence [11–14].

In the present study, a cross-sectional survey was carried out to assess the nutritional status of a sample of randomly selected children and adolescents attending UNRWA and government schools in the West Bank. The study objectives were to identify key blood and biochemical parameters to incorporate in a surveillance system; and offer evidence-based information to direct policies and nutritional interventions.

## Methods

### Sampling design

A sample of West Bank students were randomly selected from UNRWA and government schools for a 2009 cross-sectional school-based survey. The UNRWA and the government provide the bulk of free education in Palestine, serving over 85% of students [15, 16]. In order to sample the schools, the West Bank was divided into three geographical areas (north, middle, and south) each accounting for roughly one- third of the total school children population. Schools with at least 50 students (both girls and boys) in three selected grades were sampled for this study. UNRWA and government school students were stratified by region, sex, and school year (grade). The sample size required to report prevalence data (i.e., the percentage of first graders with stunting) was calculated using a one-sample proportion tests, with 80% power for a pre-set type 1 error of 5% for the main variables to be investigated. The proportion test returned a required sample size of 500 for each age group included in the study. The three age groups were as follows: first grade (mean age 6.71 years [SD 0.45]), sixth grade (mean age 11.82 years [SD 0.57]), and ninth grade (mean age 14.83 years [SD 0.61]).

The initial plan was to select six government schools and six UNRWA schools to cover the north, central, and southern regions of the West Bank, then to randomly select 42 students from each grade and class. This would have yielded 504 (42*12) students from each grade, with 252 boys and 252 girls. The plan had to be amended to accommodate the realities of UNRWA and government schools in the region.

First, students were randomly recruited from 16 rather than 4 UNRWA schools. This resulted from the selection of two camps from each region and the fact that in some UNRWA schools, the elementary school and preparatory school are separate (requiring the selection of four schools rather than two). In light of these considerations, six government and 16 UNRWA schools were selected from 1555 UNRWA and government schools. Second, it was found that some of the government schools incorrectly reported the number of students in each class; in some cases, the school did not have enough students to allow the random selection of 42 students from each grade. Consequently, only 681 students were selected from government schools (rather than 756 as initially planned). From UNRWA schools 819 students were then selected to make up the required total of 1500. One student per household was selected to avoid clustering.

For each study index, two measurements of the standing height and weight (lightly dressed, shoes removed) were taken by trained field workers on visits to the participating schools. A third measurement was taken if the difference between the two measurements was greater than 10%. Mothers were invited to attend their children’s schools on the day of the visit and were asked to provide information regarding socio-demographic characteristics. In the case of first grade students, mothers were asked to complete the student survey on behalf of their children. Sixth and ninth grade students were asked to complete a survey regarding their physical activity and the number of hours spent watching TV, among other variables.

### Laboratory measurements

Blood (5 mL) was obtained by venipuncture. To check validity of test results, two types of quality assurance program were used, internal and external quality control. Internal quality control was carried out for all lab tests, where three different levels of control were used with every batch of samples analyzed. Assays were externally validated for vitamin B12, ferritin, and folic acid levels using control quality assurance samples provided by the Centers for Disease Control and Prevention (CDC). The CDC - External Quality Assurance (EQA) program is a standardization program designed to provide laboratories with an independent assessment of their analytical performance. The blind samples were sent by the CDC-EQA program to Ramallah. A complete blood cell count was performed with a Fully Automated Hematology Analyzer by Cell Dyne 1700an. Thyroid hormones, vitamin B12, folate, and ferritin were determined using a Chemiluminesence assay using ADVIA Centaur USA, by Siemens. C-reactive protein (CRP) was determined by the ELISA technique using commercially available kits (Quantikine Human, R&D Systems, Minneapolis, MN). Zinc content was measured using atomic absorption spectrophotometer blood. Lead was determined in a randomly selected 50% of the samples using Leadcare Analyzer, ESA Biosciences. A capillary tube was used to draw a small amount of blood (approximately two drops) and deposited in the Leadcare system. Leadcare controls were done for each test kit.

In addition to the internal quality control procedures, we took the following measures in examining Fe, Zn, and lead:

- The venipuncture site was cleaned with alcohol.
- A closed-tube vacuum system was used to avoid mineral contamination.
- Special metal-free blood containers were used to minimize the potential for sample contamination by any outside sources of minerals.
- Blood was drawn in a BD Royal Blue with K2 EDTA Vacutainer tube (Supply T183).
- Lead blood specimens were drawn in a BD Tan with K2 EDTA, lead only (EDTA) Vacutainer tube (Supply T615).
- All tubes were kept in dark cool boxes (0–4 °C) and transported to the central lab.

For quality control, double data entry was used for all lab tests, in addition to survey data. Students with elevated CRP were excluded from ferritin and zinc analyses.

### Variables

***Low Mean Corpuscular Volume (MCV)*** was defined as MCV < 75 fL.

***Iron deficiency*** was defined as serum ferritin below 15 ng/ml based on WHO guidelines [17].

***Anemia:*** Based on WHO guidelines, anemia was defined as hemoglobin (Hb) below 11.5 g/dl for children 5–11 years; below 12 g/dl for children 12–14 years; and girls ≥15, and below 13 g/dl for boys ≥15 years [17].

***Iron deficiency anemia*:** was defined as having both anemia and iron deficiency.

***Serum folate:*** levels of 7–20 μg/L were defined as normal; levels of < 3.1 μg/L were defined as very low, levels of < 7.0 μg/L) were defined as low, and levels of > 20 μg/L were defined as high [18].

***Iodine deficiency***: iodine deficiency was defined by a low thyroxine which was determined according to the manufacturer’s instructions as tri-iodothyroxine (FT3) < 2.3 pg/ml, thyroxine hormone (FT4) < 0.89 g/dl, and thyroid-stimulation hormone (TSH) >  5.5 uIU/ml.

***Vitamin B12*:** levels were defined as B12 deficient (< 221 pg/ml) and marginal (< 300 pg/ml) [19].

***Serum Zinc*:** low levels were defined as < 65 μg/dl [20].

***C-reactive protein (CRP*)**: Elevated levels were defined > 11 mg/l [21].

***Lead***: High levels were defined as > 10 μg/dl [22].

### Statistical analysis

SAS (SAS Institute, Cary, NC) software was used to analyze the dataset of 1484 subjects. Means and percentages were used to describe the characteristics of the study sample. Chi-square test was performed to examine differences in micronutrient deficiency by gender, school affiliation, and grade level. Tests of significance were two-sided with *p*-value ≤0.05. The effect of fortification on the prevalence of micronutrient deficiencies was evaluated based on previous reports and an earlier baseline study in Hebron/West Bank in 2005 [23].

## Results

### Study sample

Data were obtained from 1484 (99%) of 1500 students; males accounted for 49.7% of the study population. The response rate for blood samples from the students was 98%. Baseline characteristics of the study sample are published in an earlier study [24]. Forty-six percent of the students were from government schools.

### Micronutrient status

**Fig. 1:**
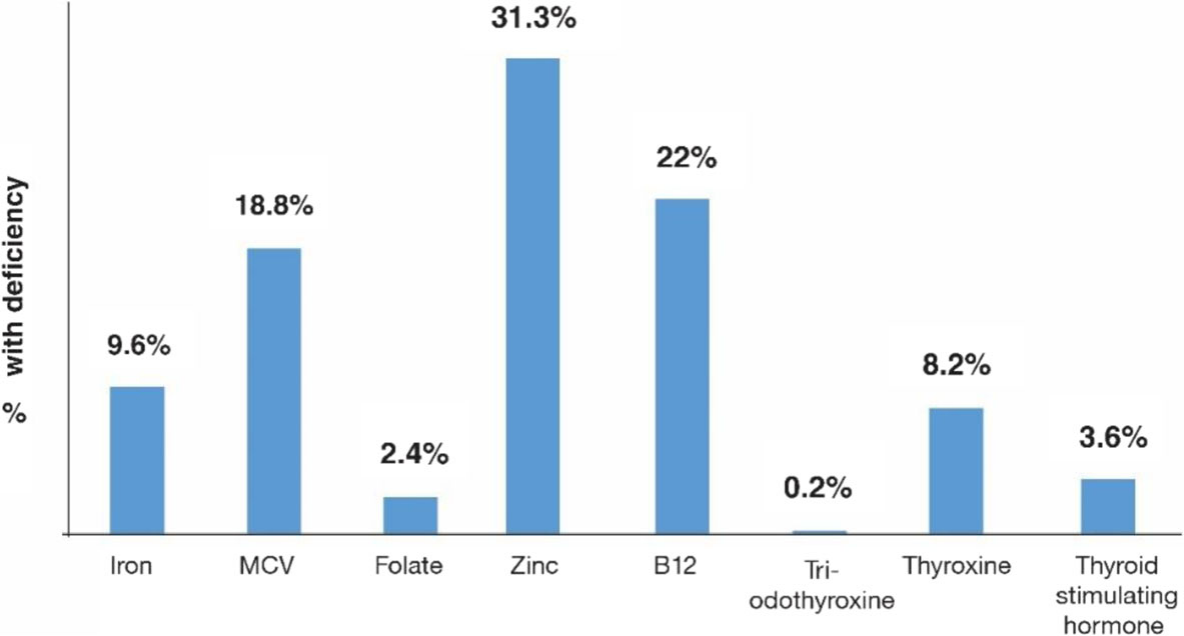
Prevalence of low micronutrient levels in the study population

The micronutrient status of the study sample is presented in Fig. 1. The students with elevated CRP (2.6% of total students) were excluded from the ferritin and zinc analyses. There were gender and grade level differences in most micronutrient levels (Table 2).

**Table 2:** Micronutrient status of the study sample by gender and grade level, (*N* = 1484), 2009

	% with Deficiency						
	First Grade		Sixth Grade		Ninth Grade		Total
Mean age (SD) in years	**6.7 (0.5)**		**11.8 (0.6)**		**14.8 (0.6)**		
	**Male**	**Female**	**Male**	**Female**	**Male**	**Female**	
**MCV**^a^	31.3	16.9	17.1	16.2	14.9	13.5	18.8
**Folate**							
- (< 3.1 μg/L)	0	0	0	0.4	0	0.4	0.1
- (< 7.0 μg/L)	0.4	2.4	1.2	1.6	3.8	4.9	2.4
- (> 20 μg/L)	29.3	33.3	22.4	32.7	18.2	15.5	25.2
**Zinc**^**2**^	34.7	35.7	32.2	33.6	20.0	31.0	31.3
**B12**							
- Marginal^3^	26.8	37.3	41.6	37.1	46.6	39.2	40.0
- Deficient^4^	15.9	13.5	21.2	27.8	36.0	19.2	22.0
**Thyroid hormones**							
- Tri-iodothyroxine^5^	0	0	0	0.8	0.4	0	0.2
- Thyroxine hormone^6^	3.7	2.8	6.1	10.9	14.4	12.2	8.2
- Thyroid- stimulation hormone^7^	6.1	4.0	2.9	5.2	1.3	2.5	3.6

^a^MCV<75 fL, ^2^Zinc < 65 μg/dL, ^3^B12< 300 pg/mL ,^4^B12<221 pg/ml, ^5^FT3 < 2.3 pg/mL, ^6^FT4 < 0.89 g/dL. ^7^TSH> 5.5 uIU/mL

Anemia, iron deficiency, and iron deficiency anemia.

**Fig. 2:**
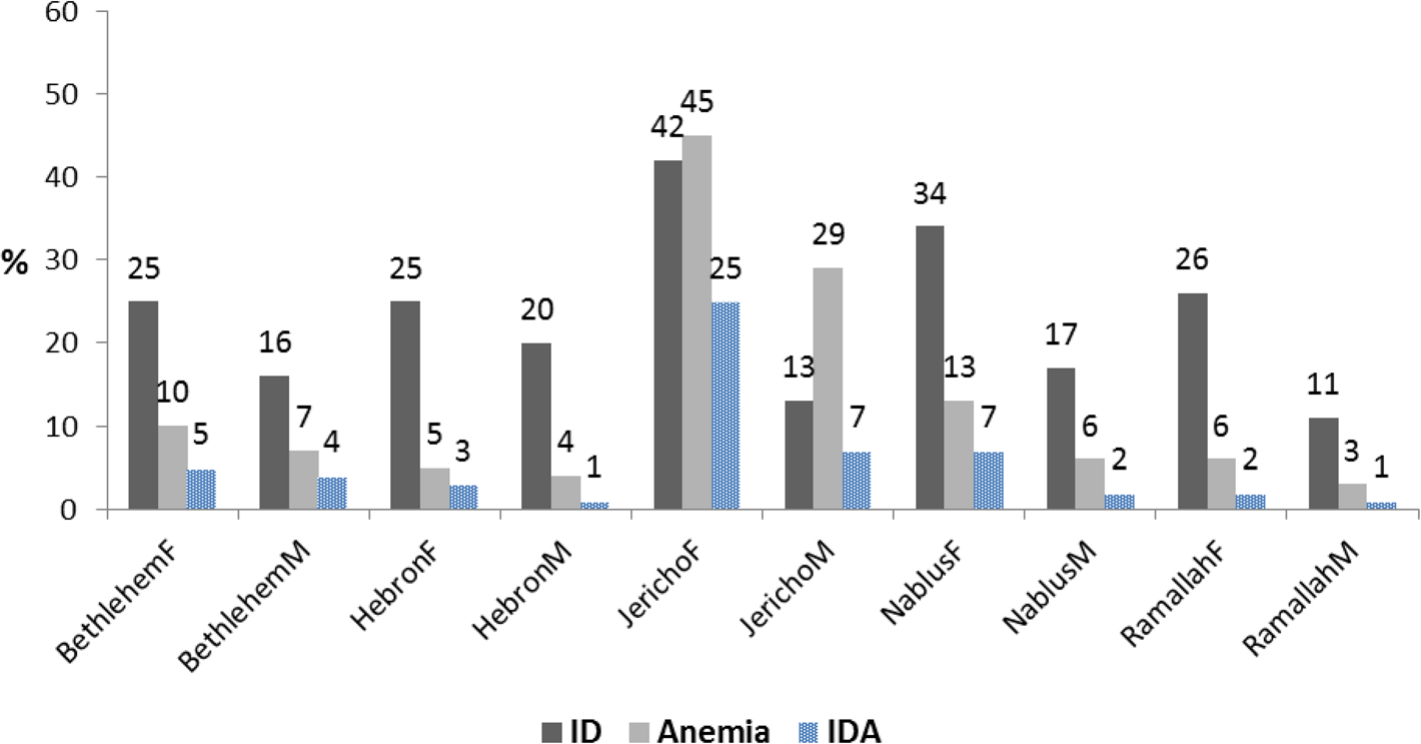
Prevalence of Anemia, Iron deficiency, and Iron Deficiency Anemia by District and Gender, *Note: F: Female, M: Male, ID = iron deficiency, IDA = iron deficiency anemia

The overall prevalence of anemia was 9.6%, with significant gender differences:12.1% girls and 7.1% boys (*p* = 0.001). There was also a significant difference by school affiliation; the prevalence of anemia was higher in UNRWA schools compared to government schools, at 13.3% vs. 5.3%, respectively (*p* = 0.0001). The prevalence of anemia was highest in Jericho and lowest in Ramallah (Fig. 2). However, there was no statistically significant differences by grade level in overall anemia (first grade: 8.9% (95% CI 6.4–11.4%), sixth grade, 8.0% (5.6–10.4%), and ninth grade, 12.1% (9.2–15.0%). Forty-two percent of children with anemia had iron deficiency.

There were significant differences in iron deficiency between girls and boys, 29.5% vs. 15.7%, respectively (*p* = 0.0001). There were also significant differences by grade level: 19.7% among first grade students, 18.7% among sixth graders, and 29.9% among ninth graders (p = 0.0001). As is the case with anemia, there were school statistically significant differences in iron deficiency, 26.3% in UNRWA schools and 18.5% in government schools (p = 0.0001).

Overall prevalence of iron deficiency anemia (IDA) was 4%: 6.2% among females and 1.8% among males. More students (5.8%) from UNRWA schools had iron deficiency anemia compared to 1.9% of those from government schools (p = 0.0001). As shown in Fig. 2, there was marked variability in iron deficiency and iron deficiency anemia by gender and district. Across all districts, except Ramallah, iron deficiency explained more than 50% of anemia among females.

### MCV levels

In general, MCV was low among a substantial number of schoolchildren; 18.8% had low MCV.

### Folate levels

Based on study findings, low folate was not a concern. On the other hand, 25.3% had elevated serum folate levels (> 20 μg/L).

### Serum zinc levels

About 31% of the study sample had zinc deficiency in a geographic distribution that approximates that of iron deficiency, with significant gender differences, 33.5% among females vs. 29.1% among males (*p* = 0.04). There was also significant variation in zinc deficiency by grade level: 35.2% among first graders, 32.9% among sixth graders, and 25.6% among ninth graders (*p* = 0.003).

### Vitamin B12 levels

Vitamin B12 levels showed statistically significant gender and grade differences, where deficiency was more common among ninth grade males (36.0% vs 19.2% among ninth grade females, *p* = 0.000).

Iodine status.

Based on study findings, there was essentially no iodine deficiency; 0.2% had deficiency in tri-iodo thyroxine, and 3.6% had deficiency in thyroid stimulating hormone.

#### Blood Lead

There were no cases of lead toxicity (blood lead > 10 microgram/dl) in the study sample.

## Discussion

Based on the levels of thyroid hormones reported in the study, iodine deficiency is seemingly no longer a nutritional problem in the West Bank. Based on the Palestinian Family Health survey of 2010, 77% of households in Palestine consumed iodized salt: 68% in the West Bank and 91% in the Gaza Strip [25]. However, despite flour fortification, main nutritional micronutrient risks for schoolchildren in the West Bank were low serum levels of iron, zinc, and vitamin B-12, and there were pockets of anemia in certain districts.

It was challenging to examine the impact of flour fortification on micronutrient status of children due to following reasons. First, there is no baseline national data on anemia and micronutrient status among children before fortification to measure impact of flour fortification. The only baseline data available on micronutrient deficiency before fortification emanates from a local study of micronutrient deficiency among 366 pairs, children (3–7 years) and his/her non-pregnant mothers (18–50 years old) whom are randomly selected from Gaza city in Gaza Strip and Hebron in the West Bank in 2005 [23]. In Hebron, 15% of children 3–7 years old had anemia [23]. In the present study, the prevalence of anemia among children in first grade in Hebron (mean age 6·71 years [SD 0·45]) was 4.7%. This may suggest that wheat fortification improved, but did not eliminate iron deficiency. Second, not all flour is fortified. According to the Palestinian Ministry of Health, in 2013, only 41% of wheat flour was actually fortified with iron in the West Bank [10]. This low percentage stems from the different sources of flour in the market, where flour from Israel and donations are not fortified.

Almost 42% (ranged between 25 and 73% based on grade and gender) of the anemia in our sample was explained by iron deficiency as measured by serum ferritin, in line with findings from research in Arab countries [5]**.** Iron deficiency among adolescent children can be explained by growth spurts and sexual maturation. Adolescent girls in particular are vulnerable to micronutrient deficiency because they need protein, iron, and other micronutrients to support the adolescent growth spurt, including increased demand for iron during menstruation.. In addition, older children may consume more outside of their home which may result in increased intake of foods with low nutritional value like salty and sweet snacks [26].

In contrast to the high prevalence of low serum ferritin, 25% of the children showed high levels of serum folate, which suggests an association with an intake of fortified wheat flour. Interestingly, the children from Bethlehem did not show as high occurrence of elevated folate levels as children from other areas. This observation may reflect higher intakes of non-fortified flour from Israel, the main source of flour in Bethlehem.

Despite fortification, low levels of B12 persist. Bioavailability of vitamin B12 is dependent on the complex production and release of proteins from the mouth and stomach, haptocorrins and intrinsic factor. Among the factors that contribute to low levels of B12 include malabsorption caused by atrophic gastritis or Helicobacter pylori infection, pancreatic or intestinal pathology, and gastric acid-reducing medications [27, 28]. Currently, we lack adequate data to explain the low levels of B12 in the present study. Shortcomings in the fortification process may have played a role.

Our study has some limitations. First, the study was conducted among children attending public schools only; government and UNRWA schools and excluded those from private schools.

However, it included most children, as about 86% of schoolchildren in the West Bank go to public schools. Second, the study examined only school children, may not apply to other population groups. However, Palestine has a young population in which 45% of Palestinian are children. Third, we used data from 10 years ago for this paper. However, our survey describes the only micronutrient data available on a random sample of children 6–12 years. Adding to that, micronutrient deficiency is still a nutrition concern among school children along with challenges of low fortification levels in fortified flour in the West Bank. Despite these limitations, the present study is unique in several aspects. In the first instance, the subjects who took part in the study were sampled from districts in the northern, central and southern parts of the country. Furthermore, the study examined micronutrient status of different age groups—as opposed to only adolescents—that were randomly selected instead of resorting to recruitment of a convenient sample. In addition to this, several indicators of nutritional status were utilized in order to broaden and deepen attributes of the participants.

## Conclusions

Our results indicate that the main nutritional micronutrient risks for schoolchildren in the West Bank continue to be low serum levels of iron, zinc, and vitamin B-12; folate levels were seemingly high; and for the whole population iodine intake appears adequate. Iron deficiency remains a public health concern among the school children, mainly 6th and 9th grade female students. With the exception of Jericho, anemia is mild in school-age children of the West Bank; although the prevalence increases in male after the 6th grade. The recommended key blood and biochemical parameters to be incorporated in the surveillance system are iron, zinc, and B12.

Several interventions are possible to combat anemia, iron, zinc and vitamin B12 deficiencies. First, anemia can be combatted by revising fortification levels through an increase in iron, zinc, and vitamin B12, for example. Consideration needs to be given to enforce laws for flour fortification and better control on smuggled unfortified flourfrom Israel and on donated flour based on Palestinian specification. Second, resources can be funneled into agricultural practices to enhance food availability and ensure access to particular foods [3]. Third, complementary measures can be undertaken, including dietary diversification and supplementation. Fourth, ministries need to collaborate with the local food industry to fortify food products, to offer healthy food products. Additionally, there is a need for nutritional intervention in adolescent girls before the onset of childbearing.

## Data Availability

The dataset is available upon reasonable request.

## Abbreviations

EMR: Eastern Mediterranean Region
UNRWA: the UN Relief and Works Agency for Palestine Refugees in the Near East
